# *Enterobacterales* Biofilm-Specific Genes and Antimicrobial and Anti-Inflammatory Biomarkers in the Blood of Patients with Ischemic Heart Disease

**DOI:** 10.3390/diagnostics14050546

**Published:** 2024-03-05

**Authors:** Agne Giedraitiene, Vacis Tatarunas, Kornelija Kaminskaite, Ugne Meskauskaite, Svitlana Boieva, Yu Ajima, Ieva Ciapiene, Audrone Veikutiene, Vaidotas Zvikas, Nora Kupstyte-Kristapone, Valdas Jakstas, Dalia Luksiene, Abdonas Tamosiunas, Vaiva Lesauskaite

**Affiliations:** 1 Institute of Microbiology and Virology, Lithuanian University of Health Sciences, Eiveniu 4, LT 50161 Kaunas, Lithuania; 2 Institute of Cardiology, Lithuanian University of Health Sciences, Sukileliu 15, LT 50103 Kaunas, Lithuania; vacis.tatarunas@lsmu.lt (V.T.); svitlana.boieva@lsmu.lt (S.B.); ieva.ciapiene@lsmu.lt (I.C.); audrone.veikutiene@lsmu.lt (A.V.); dalia.luksiene@lsmu.lt (D.L.); abdonas.tamosiunas@lsmu.lt (A.T.); vaiva.lesauskaite@lsmu.lt (V.L.); 3 Medical Academy, Lithuanian University of Health Sciences, A. Mickeviciaus 9, LT 44307 Kaunas, Lithuania; kornelija.kaminskaite@stud.lsmu.lt (K.K.); ugne.meskauskaite@lsmu.lt (U.M.); b205356@hiroshima-u.ac.jp (Y.A.); nora.kupstyte@lsmu.lt (N.K.-K.); 4 School of Medicine, Hiroshima University, 1-2-3 Kasumi, Minami-ku, Hiroshima 734-8553, Japan; 5 Institute of Pharmaceutical Technologies, Lithuanian University of Health Sciences, Sukileliu 13, LT 50161 Kaunas, Lithuania; vaidotas.zvikas@lsmu.lt (V.Z.); valdas.jakstas@lsmu.lt (V.J.)

**Keywords:** *E. coli*, bacterial DNA, biofilm, blood direct PCR, ischemic heart disease, cardiovascular diseases

## Abstract

Background: Ischemic heart disease (IHD) is the most prevalent type of cardiovascular disease. The main cause of IHD is atherosclerosis, which is a multifactorial inflammatory disease of blood vessels. Studies show that bacteria might have a significant impact on the pathogenesis of atherosclerosis and plaque rupture. This study aimed to evaluate the complexity of interactions between bacteria and the human body concerning metabolites and bacterial genes in patients with ischemic heart disease. Methods: Bacterial *16S rDNA* and *wcaF*, *papC*, and *sdhC* genes were detected in whole blood using a real-time PCR methodology. An enzyme-linked immunosorbent assay was used to measure the concentration of the LL-37 protein. An analysis of ARA in blood plasma was performed. Results: Bacterial *16S rDNA* was detected in 31% of the study patients, and the genes *wcaF* and *sdhC* in 20%. *Enterobacterales* genes were detected more frequently in patients younger than 65 years than in patients aged 65 years and older (*p* = 0.018) and in patients with type 2 diabetes (*p* = 0.048). Concentrations of the human antimicrobial peptide LL-37 and 12S-HETE concentrations were determined to be higher if patients had *16S rDNA* and biofilm-specific genes. Conclusions: The results of this study enhance the understanding that *Enterobacterales* bacteria may participate in the pathogenesis of atherosclerosis and IHD. Bacterial DNA and host metabolites in higher concentrations appear to be detected.

## 1. Introduction

Cardiovascular diseases (CVD) remain the main cause of death in Western countries, and ischemic heart disease (IHD) is the most prevalent among CVD. It was estimated that around 126 million people, who make up approximately 1.7% of the world’s population, have IHD [1]. In Central and Eastern European countries, an increase in IHD morbidity, compared to Northern, Southern, and Western Europe, is observed [2]. The standardized rate of deaths from IHD of 5362 deaths per million inhabitants made Lithuania a leader with the highest mortality rate among the European Union (EU) Member States in 2017 [3].

The main cause of IHD is atherosclerosis. Atherosclerosis is an inflammatory lipoprotein-driven multifactorial disease causing atherosclerotic plaque development and coronary blood flow reduction. If a rupture or erosion of a plaque’s fibrous cap occurs in coronary arteries, it might result in unstable angina or myocardial infarction. The complexity of this condition has led to great concern in finding novel tools for the early diagnosis or prognosis of atherosclerosis and IHD [4,5,6,7].

One of the possible factors for an early diagnosis of IHD could be a biomarker of atherosclerosis progression, the genetic material of certain species of bacteria [8]. Numerous studies have shown that various bacteria and viruses have a direct impact on the vascular endothelium [9,10]. Pathogens are found in the atherosclerotic plaque, and they are harbored in the latent state [11]. A lipopolysaccharide of *Escherichia coli* (*E. coli*) was detected in atherosclerotic plaques, and possibly it is related to the damage and rupture of plaques via Toll-Like Receptor 4 (TLR4)-mediated oxidative stress [12]. Zdimal and Davies, 2022, demonstrated that if a biofilm is exposed to an elevated concentration of free iron, it may undergo dispersion contributing to the weakening of arterial tissues and destabilization of the atherosclerotic plaque [13].

The study demonstrated that bacteria might form a biofilm within human carotid arterial plaques [14]. Microbial cells within biofilms tolerate up to 100–1000 times higher concentrations of antibiotics and enhance bacterial survival in the human body, including the bloodstream [15,16]. More than 100 genes have been encountered encoding biofilm in *E. coli* [17,18]. The *wcaF* gene encodes an acetyltransferase associated with the polysaccharide colanic acid’s synthesis. Colanic acid is an extracellular polysaccharide needed for the formation of the complex three-dimensional structure of *E. coli* biofilms [19]. The P-fimbriae, encoded by the pap operon, is responsible for adhesion and is found in many UTI *E. coli* strains. The *papC*-encoded protein forms pores in the outer membrane of *E. coli* and is responsible for the transportation of protein pilin subunits [20]. The *sdhC* gene encodes one of the four subunits of the inner membrane protein succinate dehydrogenase, which is involved in the Krebs cycle [21]. All three genes *wcaF*, *papC*, and *sdhC* are located on the *E. coli* chromosome and encode proteins directly involved in biofilm formation.

The *16S* rRNA gene is a part of the prokaryotic ribosome 30S subunit and it is coded in the genomes of all bacteria. The *16S* rRNA gene sequencing has been used for the identification, classification, and quantitation of microbes for decades [22]. It may also allow the identification of new and non-cultured bacteria [23], as *16S* rRNA is highly conserved and specific. It plays a central role in phylogenetic research, as it is sustained between different species of bacteria and archaea [24].

The detection of bacteria in the blood flow is usually associated with a low sensitivity in the presence of extremely low concentrations and time-consuming methodology [25]. The inhibition of PCR amplification and interference with the detection of fluorescence slow down bacterial detection and identification [26]. New enzymes used in the polymerase chain reaction, which have been developed during recent years, allow researchers to overcome these weak points of research and ensure the successful performance of the bacterial gene amplification directly from patient blood (without the extraction of DNA before amplification in classic PCR).

LL-37 is a cathelicidin-related antimicrobial peptide (CRAMP). It plays a crucial role in the immune response to bacterial infections and is actively involved in regulating inflammatory processes. CRAMP enhances the chemotactic responsiveness of bone marrow and progenitor cells [27]. A study shows that decreased LL-37 might be associated with myocardial infarction [28,29], while a high LL-37 level predicts lower major adverse cardiovascular events after ST-segment elevation myocardial infarction [30,31].

Stimulated immune cells may produce significant amounts of arachidonic acid metabolites such as 12-HETE, 15-HETE, PGE_2_, and LTB4. These compounds could be an early inflammatory signal for the initiation of the immune system [32].

Thus, this study aimed to assess the complexity of interactions between bacteria and the human body concerning metabolites and bacterial genes in patients with ischemic heart disease. Moreover, concentrations of the protein LL-37 and arachidonic acid (ARA) metabolites produced by a host in the immune response to ischemic heart disease were measured, and associations between all parameters were determined.

## 2. Materials and Methods

### 2.1. Study Population and Inclusion Criteria

The study included 75 randomly selected patients with ischemic heart disease (39 men and 36 women), and 75 healthy persons (39 men and 36 women) as a control group. All patients were hospitalized due to ischemic heart disease at the Department of Cardiology at the Hospital of Lithuanian University of Health Sciences Kaunas Clinics in Kaunas, Lithuania, from 2013 to 2017, and were treated with dual antiplatelet therapy (DAPT) of clopidogrel, or ticagrelor and aspirin. Hormone anti-inflammatory medications were not prescribed to the patients.

The exclusion criteria of patients were conditions leading to an increased activity of coagulation (malignant neoplasia and severe inflammation [C-reactive protein level > 100 mg/L] or patients who had undergone antibiotic therapy due to infection, patients diagnosed with atrial fibrillation or pericardial diseases, significant structural heart disease such as valvular heart disease), cardiogenic shock or hypovolemia, and the refusal of a patient to take part in this study.

The patient population’s clinical data and blood samples were collected during the SEN-09/2015 study [33,34].

The inclusion criteria for the control group: (1) Control subjects were followed up for 10 years for CVD mortality events and health conditions/diseases after inclusion in the international study Health, Alcohol and Psychosocial Factors in Eastern Europe (HAPIEE), conducted in Kaunas (Lithuania) [35]; (2) None of the participants had a cardiovascular disease, stroke, or diabetes during the follow-up period. The mean age of healthy subjects was 67.7 years (minimum age—45, maximum age—72).

**Figure 1:**
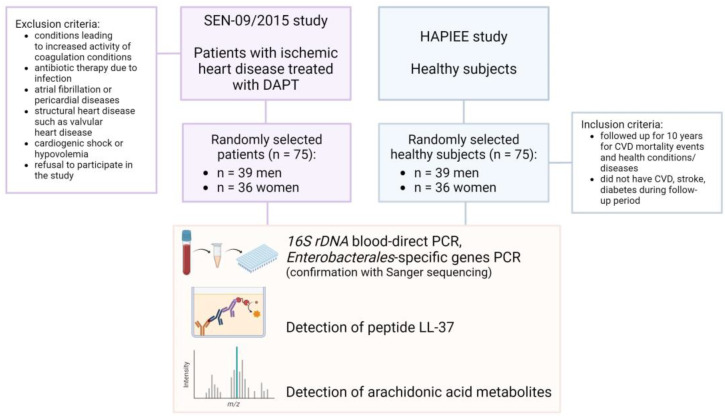
Flowchart. Study design including exclusion and inclusion criteria and performed procedures.

The study design is presented in Figure 1.

### 2.2. Preparation of Blood Samples

Venous blood sampling of patients was carried out by a routine venipuncture procedure using plastic tubes containing 3.2% sodium citrate anticoagulant (BD Vacutainer, Franklin Lakes, NJ, USA). All blood samples were stored at −20 °C. It is known that PCR-inhibitory compounds in human blood can considerably reduce the sensitivity of the PCR assay [36]. The blood protein hemoglobin adversely affects DNA polymerase activity and inhibits amplification and fluorescence signaling; immunoglobulin G disrupts amplification of the first PCR cycles by binding to single-stranded DNA [37]. Red blood cells naturally fluoresce across multiple wavelengths, which span the emission and excitation spectra of many commonly used fluorescent reporters and dyes such as *SYBR Green I*, and *Midori Green*, making the endogenous fluorescence difficult to distinguish [38]. One microliter of whole blood was taken for the assay. The mixture of blood and a buffer, the composition of which was developed in our laboratory, were gently mixed and heated for 5 min at 98 °C, then centrifuged for 2 to 4 s (2700 rpm). The amplification and detection of bacterial genes were performed using a QuantStudio 3 Real-Time System instrument (Thermo Fisher Scientific, Waltham, MA, USA). A blood direct PCR (BD-PCR) using genomic DNA without its extraction was performed directly from 1 µL of treated whole blood using specific primers. A mix of primers was prepared to amplify bacterial *16S rDNA*. Primer sequences (Table 1), the primer mixture (Table 2), and reaction parameters (Table 3) were adopted from Barghouthi S. A. et al., 2011 [39], And oligonucleotide primers used for the amplification of wcaF, papC, and sdhC were adopted from the following references [31,32,40] (Table 4). The reaction was performed in standard 96-well real-time PCR plates. Thermal cycling program parameters used in blood direct PCR are shown in Table 3 and Table 5. Amplicon size was verified in 2% agarose gel. Bacterial DNA from selected strains was used as a positive control.

### 2.3. Extraction of Bacterial DNA

A reference *E. coli* ATCC 25922 strain was used to prepare a bacterial suspension. A few colonies of one-day *E. coli* bacteria were mixed with 700 µL nuclease-free water, then the suspension was vortexed and centrifuged for 2–4 s (2700 rpm). Bacterial cell wall structures were disrupted mechanically, and high-fidelity DNA polymerase (Thermo Fisher Scientific, Vilnius, Lithuania) was used for the gene amplification; no further DNA purification was required.

### 2.4. Confirmation of Bacterial DNA with Sanger Sequencing

The purification of randomly selected PCR products for bacterial DNA sequencing from TBE-buffered agarose gel was performed using the Zymoclean™ Gel DNA Recovery Kit (Zymo Research, Irvin, CA, USA). DNA sequencing was conducted by using the Sanger sequencing method. The interpretation of sequencing chromatograms was carried out with Chromas (Technelysium, Brisbane, Queensland, Australia).

### 2.5. Detection of LL-37

The sandwich enzyme immunoassay (Enzyme-linked Immunosorbent Assay Kit “Human Antibacterial Peptide LL-37 ELISA Kit” (Cusabio Technology LLC, Houston, TX, USA) was used to measure LL-37 in human plasma. All reactions were performed following the manufacturer’s instructions. LL-37 was detected by measuring optical density at 450 nm using a microplate reader Stat Fax 4200 (Awareness Technologies, Palm City, FL, USA). The concentration of the LL-37 peptide was calculated using a standard calibration curve. The calibration curve was performed using Curve Expert 1.4 (Hyams Development, Huntsville, AL, USA).

### 2.6. Detection of ARA Metabolites in Blood Plasma

The concentrations of ARA metabolites in blood plasma were measured as described above [41]. The extraction and preparation of the samples for analysis were conducted at the Laboratory of Molecular Cardiology. The analysis of ARA metabolites in blood plasma was performed at the Institute of Pharmaceutical Technologies of the Lithuanian University of Health Sciences.

### 2.7. Statistical Analysis

Statistical analysis was performed with IBM SPSS Statistics V27 software (IBM Corp., Foster City, CA, USA). The Shapiro–Wilk test was used to check the normality of the variable’s distribution. Pearson’s *χ*^2^ or Fisher′s exact test was used for categorical variables. The results were considered statistically significant when *p* < 0.05.

## 3. Results

Bacterial *16S rDNA* was detected in 31% (n = 23/75) and genes specific for *Enterobacterales wcaF* and *sdhC* but not the *papC* were detected in 20% (n = 15/75) of the whole-blood samples of the study patients. The highest percentage of *Enterobacterales*-specific genes detected in the analyzed samples was 13.3% of the gene *wcaF* (n = 10/75), followed by 8% of the gene *sdhC* (n = 6/75). One of the patients had both the *wcaF and sdhC* genes. The differences in gene prevalence by sex were not significant due to the small sample size (Table 6). The *16S* rRNA gene, a molecular marker for the identification of bacterial species, was not detected in any of the control subjects. Further analysis of the healthy group was not performed.

### 3.1. Bacterial DNA Sequencing Results

Sanger sequencing was performed for the PCR product of a blood sample with the *wcaF* gene positive. Analysis of the resulting sequence in the BLAST program corresponded to *Escherichia* spp. bacterium by 93.8%. Sequencing of the PCR product of a blood sample with the *sdhC* gene detected by RT-PCR showed the *sdhC* gene encoded by bacteria of the genus *Serratia* or *Escherichia* (90.5% and 88.6%, respectively).

### 3.2. Enterobacterales Genes in Patient Blood

The distribution of *Enterobacterales* genes may vary according to the patient’s gender and age. Bacterial genes tended to be detected more frequently in the blood of men than women (*p* = 0.086) (Appendix A). Patients younger than 65 years old more frequently had bacterial genes detected in their blood than patients aged 65 years and older (*p* = 0.018). Additionally, bacterial genes were more frequently detected in patients with type 2 diabetes (*p* = 0.048) (Appendix A). The results with *16S rDNA* were not significant.

### 3.3. Protein LL-37 Concentration and Bacterial Genes

The concentrations of LL-37 were higher in patient blood with *16S rDNA* and biofilm-specific genes (Table 7).

### 3.4. Concentration of Arachidonic Acid Metabolites and Presence of Enterobacterales Genes in Blood Samples

Patients with *Enterobacterales* genes in their blood had a higher concentration of 12S-HETE and 5S-HETE than patients without bacterial genes (respectively, *p* = 0.046 and *p* = 0.077) (Table 8). The results with *16S rDNA* were not significant.

### 3.5. LL-37 and Arachidonic acid Metabolites

A positive correlation of LL-37 was observed with 5S-HETE (r = 0.367, *p* = 0.015) when a trend was determined between LL-37 and 12S-HETE (r = 0.307, *p* = 0.048) in blood plasma.

## 4. Discussion

This study reports that bacterial *16S rDNA* was detected in one-third of the venous blood samples of patients with IHD (30.5%). The presence of *16S rDNA* was not significantly associated with IHD, diabetes, or the other factors or conditions analyzed in our study. The healthy population sample tested negative for bacterial *16S rDNA* and biofilm-encoding genes. The presence of DNA, a marker of bacterial translocation and a potential biomarker, was reported by other studies in patients with cirrhosis [42], cardiovascular disease [43], type 2 diabetes [44], psoriatic arthritis [45], and atherosclerosis [46]. Bacteria might play a role in the development and progression of atherosclerosis by the formation of biofilms within arterial plaques and the rupture of unstable plaques [14,47]. A plaque’s rupture leads to a life-threatening atherothrombotic lesion, occlusion of the coronary artery, and myocardial ischemia. However, the mechanism of IHD pathogenesis which involves the formation of bacterial biofilms is still not fully understood. There is a big shortage of knowledge on how bacterial fragments accumulate in different parts of the host’s body. One of the possible ways is bacterial biofilm formation [48]. We determined that biofilm-specific genes were more prevalent among patients younger than 65 years old and patients with diabetes. Anhe et al., 2020, determined that plasma samples of individuals with diabetes were enriched with *Enterobacterales* [49]. Possibly, the increase in the bacterial count of some specific genera might be related to the use of antidiabetic drugs, as *E. coli* proliferation is increased due to insulin administration under in vitro conditions. Insulin may serve as a signal molecule for the formation of bacterial biofilms [50,51]. About 17% of the study patients had *Enterobacterales* genes in their blood: 13.3% had the gene *wcaF*, followed by 8% with the gene *sdhC*. One of the patients had both the *wcaF and sdhC* genes. The gene *wcaF* encodes an acetyltransferase associated with the polysaccharide colanic acid’s synthesis [19]. Prospective studies have shown that exopolysaccharides’ (alginate and colanic acid) synthesis is induced upon attachment of bacteria to the surface [52], but at the same time, the biofilm formation-associated gene *wcaF* might be regulated by the quorum sensing *E. coli* regulators B and C [40]. Thus, by regulating exopolysaccharides, the gene *wcaF* allows control of the *E. coli* biofilm formation [53]. Importantly, the P-fimbriae, encoded by the pap operon, is responsible for adhesion and is found in many UTI *E. coli* strains [20,54]. It is worth noting that the *papC* gene might be associated with amoxicillin resistance [20]. Moreover, the *sdhC* gene encodes one of the four inner membrane protein subunits of succinate dehydrogenase, which is involved in the Krebs cycle [21]. A lower prevalence of the *papC* gene was determined in ciprofloxacin-resistant uropathogenic *E. coli* than in their susceptible counterparts [55].

**Figure 2:**
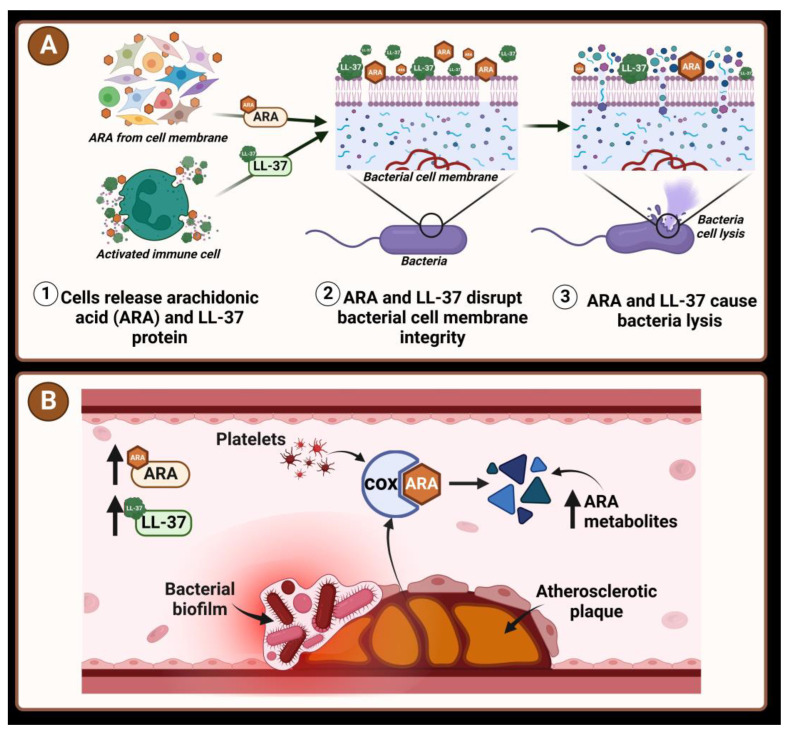
The scheme shows possible mechanisms of interaction between bacteria and host metabolites arachidonic acid (ARA) and LL-37. (**A**) ARA and LL-37 attack bacterial membranes; (**B**) Atherosclerosis in blood vessels, bacteria in biofilms, high ARA metabolites produced by COX, high LL-37 levels.

It is known that the bacterial cell wall components peptidoglycan and lipopolysaccharides (LPS) alter immune and glucose homeostasis in the host, and bacterial translocation possibly influences the host’s metabolism [56]. Also, the production of immune response signals (such as the synthesis of CRAMP peptides or arachidonic acid derivatives) takes place during the invasion of the host organism [32,57]. This research showed that patients with bacterial genes in blood plasma tend to have higher 12S-HETE and LL-37 concentrations than patients with no *Enterobacterales* genes detected. Certain host-derived metabolites of lipids could have antimicrobial activity via membrane interactions. It was determined that arachidonic acid (ARA) might reduce the virulence of certain strains of *E. coli* [58], be toxic to certain bacteria, and be able to kill *Staphylococcus aureus* [59]. The inhibition of the production of cyclooxygenase-derived compounds of ARA induces the accumulation of precursors, especially ARA [56]. The results of our study showed that the concentration of 12S-HETE, a derivative of ARA, was higher in patients with detected biofilm-forming genes than in patients with no bacterial genes. The bio-active molecular 12S-HETE participates in the host metabolism of glucose [60] and inflammation [61]. In addition, our results showed that the cathelicidin LL-37 concentration was higher in patients with *16S rDNA* and biofilm-specific genes in their blood than in patients without these bacterial genes. The antimicrobial host defense peptide cathelicidin LL-37 is a part of the innate immune response in humans. It is expressed during infection and kills bacteria via membrane interactions [62]. Studies have shown that expression of LL-37 is increased up to six-fold in atherosclerotic lesions [63]. The peptide LL-37 is produced by cells of the immune system: neutrophils, monocytes, macrophages, and epithelial cells [64]. Hypothetical mechanisms of action of bacteria and the host metabolites in the pathogenesis of IHD are shown in Figure 2.

Assessing bacterial genes such as biofilm-encoding genes or *16S* ribosomal-ribonucleic acid might be useful in diagnostics or the prediction of non-infectious diseases such as cardiovascular diseases. Rigorous referral pathways to perform molecular tests may result in significant savings [65]. Genetic screening for some specific genes and testing individuals in advance even if they are asymptomatic, but have a family history of cardiovascular diseases, might help to reduce healthcare costs, personalize treatment, and reduce the number of interventions. Overall, it might improve the patient’s quality of life and survivability [66]. To sum up, it is essential to consider introducing molecular methods for bacterial gene detection/determination in clinical practice that would allow clinicians to obtain more detailed information about the disease and more accurately diagnose it.

## 5. Conclusions

Bacterial biofilm-specific DNA was more prevalent in IHD patients younger than 65 years of age and in patients with diabetes. Concentrations of the human antimicrobial peptide LL-37 in blood were determined to be higher if patients had *16S rDNA* and biofilm-specific genes. Additionally, the concentration of the ARA metabolite 5S-HETE correlated with the LL-37 protein concentration. Thus, the results of this study enhance our understanding that *Enterobacterales* bacteria may participate in the pathogenesis of atherosclerosis and IHD. The described biomarkers might be useful for the identification of patients who might be at risk of atherosclerotic plaque rupture and IHD. Nevertheless, further studies are needed to elucidate the specific role of the host and microorganism metabolites in the progression and development of atherosclerosis.

## Figures and Tables

**Table 1:** *16S rDNA* primers used in blood-direct PCR.

*16S rDNA* Primer	Primer Sequence	Oligomer Location
F3 (Forward)	5′-GATACCCTGGTAGTCCA-3′	753–769
R3 (Reverse)	5′-TGGACTACCAGGGTATC-3′	769–752
F4 (Forward)	5′-CCGCCTGGGGAGTACG-3′	840–856
R4 (Reverse)	5′-CGTACTCCCCAGGCGG-3′	856–840
F5 (Forward)	5′-CCTACGGGAGGCAGCAG-3′	326–343
F6 (Forward)	5′-GCAGCCGCGGTAATAC-3′	481–497
R1b (Reverse)	5′-TACCTTGTTACGACTTC-3′	1468–1451

**Table 2:** Primer mixtures used for *16S rDNA* blood direct PCR.

General Mixtures	Oligomers Used in Mixtures
Reaction one	F5, F6, R1b
Reaction two	F5, F6, R3
Reaction two (b)	F5, F6, R3, R4
Reaction three	F3, F4, R1b

**Table 3:** PCR program for *16S rDNA*.

Step	Temperature (°C)	Time	Cycles
Initialization	95	5 min	1
Denaturation	95	30 s	35
Annealing	48	30 s	
Elongation	50	30 s	

**Table 4:** Oligonucleotide primers used for the amplification of *wcaF*, *papC*, and *sdhC*.

Gene	Primer	Primer Sequence	Amplification Product Size (bp)	Reference
*wcaF*	Forward	5′-TCTCGGTGCCGAAAGGGTTC-3′	236	[40]
	Reverse	5′-ATTGACGTCATCGCCGACCC-3′		
*papC*	Forward	5′-GACGGCTGTACTGCAGGGTGTGGCG-3′	328	[31]
	Reverse	5′-ATATCCTTTCTGCAGGGATGCAATA-3′		
*sdhC*	Forward	5′-CGCCAGCCGCCCAGCACAG-3′	285	[32]
	Reverse	5′-GGTATGGAAGGTCTGTTCCGTCA GATTGGTATTTACAGCCC-3′		

**Table 5:** PCR program for *wcaF*, *papC*, and *sdhC* amplification.

Step	Temperature (°C)	Time	Cycles
Initialization	98	5 min	1
Denaturation	98	5 s	40
Annealing	58	15 s	
Elongation	72	30 s	

**Table 6:** Prevalence of *16S rDNA* and genes responsible for biofilm formation in blood samples of women (n = 36) and men (n = 39).

Genes	Gene Detection	Blood Samples from Female Patients, n (%)	Blood Samples from Male Patients, (n) %	*p*-Value
*16S rDNA*	Detected	8 (22.2)	15 (38.5)	0.142
	Not detected	28 (77.8)	24 (61.5)	
*wcaF*	Detected	3 (8.3)	7 (17.9)	0.176
	Not detected	33 (91.7)	39 (82.1)	
*papC*	Detected	0 (0)	0 (0)	–
	Not detected	36 (100)	39 (100)	
*sdhC*	Detected	1 (2.8)	5 (12.8)	0.089
	Not detected	35 (97.2)	34 (87.2)	

Note: *p*—significance level.

**Table 7:** Presence of bacterial genes and concentration of LL-37 in patient blood.

	n (%)	LL-37 Concentration (ng/mL) Median (Min–Max)	*p*
*16S rDNA*			
Present	18 (30.5)	4 (1.3–11.8)	0.014
Absent	41 (69.5)	2.8 (0.8–9.2)	
Total	59 (100)	2.9 (0.8–11.8)	
**Biofilm-associated genes**			
Present	10 (16.9)	4.4 (1.3–8.4)	0.03
Absent	49 (83.1)	2.8 (0.8–11.8)	
Total	59 (100)	2.9 (0.8–11.8)	

**Table 8:** The concentration of arachidonic acid metabolites in patients with *Enterobacterales* genes in their blood.

Variable	*Enterobacterales* Genes Detected	*Enterobacterales* Genes Not Detected	*p*-Value	Total
5S-HETE (ng/mL) (min–max)	21 (6.4–68.1)	12.7 (1.5–57)	0.077	13.7 (1.5–68.1)
9-HETE (ng/mL) (min–max)	3.9 (1.6–14)	3.2 (0.1–20.7)	0.207	3.3 (0.1–20.7)
12S-HETE (ng/mL) (min–max)	6.4 (1–20.9)	1.9 (0.2–30.5)	0.046	3 (0.2–30.5)
15S-HETE (ng/mL) (min–max)	5.3 (3.4–80.9)	8.6 (3–289)	0.446	7.2 (3–289)

## Data Availability

The data presented in this study are available on request from the corresponding author. The data are not publicly available, due to the next work.

## Appendix A

**Table A1:** Patient baseline characteristics according to the detection of bacterial genes.

Factor	*Enterobacterales* Genes Detected	*Enterobacterales* Genes Not Detected	Pearson *χ*^2^ *p*-Value or *p*-Value between Medians	Total
**Sex**				
Males n (%)	11 (28.2)	28 (71.8)	3.419 *p* = 0.086	39 (100)
Females n (%)	4 (11.1)	32 (88.9)		36 (100)
**Antropometric parameters**				
Less than 65 years old n (%)	10 (34.5)	19 (65.5)	6.198 *p* = 0.018	29 (100)
65 years and older n (%)	5 (10.9)	41 (89.1)		46 (100)
Age in years median (min–max)	64.3 (44.5–79.3)	70.1 (42.5–87.5)	*p* = 0.039	68.9 (42.5–87.5)
Patient weight in kg median (min–max)	88 (53–113)	78 (45–134)	*p* = 0.378	80 (45–134)
Body weight index in kg/m^2^ median (min–max)	26.9 (18.1–45.3)	27.7 (17.3–49.8)	*p* = 0.624	27.3 (17.3–49.8)
Waist circumference in cm median (min–max)	92 (67–110)	92 (70–132)	*p* = 0.886	92 (67–132)
**Left ventricle ejection fraction**				
Left ventricle ejection fraction in % median (min–max)	45 (20–55)	50 (25–55)	*p* = 0.668	49 (20–55)
**ST-elevation MI**				
STEMI n (%)	4 (14.3)	24 (85.7)	0.912 *p* = 0.388	28 (100)
NSTEMI n (%)	11 (23.4)	36 (76.6)		47 (100)
**MI in anamnesis**				
First n (%)	11 (19.3)	46 (80.7)	0.073 *p* = 0.747	57 (100)
Recurrent n (%)	4 (22.2)	14 (77.8)		18 (100)
**Hypertension**				
Yes n (%)	7 (15.2)	39 (84.8)	1.701 *p* = 0.241	46 (100)
No n (%)	8 (27.6)	21 (72.4)		29 (100)
**Diabetes**				
Yes n (%)	7 (36.8)	12 (63.2)	4.511 *p* = 0.048	19 (100)
No n (%)	8 (14.3)	48 (85.7)		56 (100)
**Smoking**				
Smokers n (%)	3 (16.7)	15 (83.3)	0.079 *p* = 1	18 (100)
Non-smokers n (%)	11 (19.6)	45 (80.4)		56 (100)
**Concomitant drug users, n (%)**				
Antidiabetic drugs	6 (35.3)	11 (64.7)	3.214 *p* = 0.091	17 (100)
Insulin	3 (42.9)	4 (57.1)	2.521 *p* = 0.138	7 (100)
ACE inhibitors	15 (21.4)	55 (78.6)	1.339 *p* = 0.576	70 (100)
Angiotensin II receptor antagonists	1 (25)	3 (75)	0.066 *p* = 1	4 (100)
Beta adrenoblockers	15 (21.1)	56 (78.9)	1.056 *p* = 0.578	71 (100)
Statins	15 (20.3)	59 (79.7)	0.253 *p* = 1	74 (100)
Calcium Channel Blockers	1 (20)	4 (80)	0.000 *p* = 1	5 (100)
Diuretics	4 (25)	12 (75)	0.318 *p* = 0.725	16 (100)
Proton pump inhibitors	2 (50)	2 (50)	2.377 *p* = 0.176	4 (100)
**Blood test parameters**				
Platelet count (×10^9^/L) median (min–max)	224 (94–926)	220 (112–1000)	*p* = 0.632	222 (94–926)
WBC (×10^9^/L) median (min–max)	9.43 (5.3–19.9)	8.67 (4.34–16.9)	*p* = 0.251	8.77 (4.34–19.9)
C-reactive protein (mg/L) median (min–max)	1.68 (1–249.5)	6.33 (1–275.6)	*p* = 0.185	5.88 (1–275.6)
Creatinine (µmol/L) median (min–max)	89 (73–303)	86.5 (54–262)	*p* = 0.672	87 (54–303)
Hemoglobin (g/L) median (min–max)	134 (107–153)	130.5 (89–160)	*p* = 0.543	132 (89–160)
Platelet aggregation after induction with ADP (%)	18 (6–59)	18.5 (6–68)	*p* = 0.791	18 (6–68)

MI—myocardial infarction, STEMI—ST-elevation myocardial infarction, NSTEMI—a non-ST-elevation myocardial infarction, ACE inhibitors—angiotensin-converting enzyme inhibitors, WBC—white blood cells, ADP—adenosine diphosphate.
